# Bioactive Glass Applications in Dentistry

**DOI:** 10.3390/ijms20235960

**Published:** 2019-11-27

**Authors:** Hans Erling Skallevold, Dinesh Rokaya, Zohaib Khurshid, Muhammad Sohail Zafar

**Affiliations:** 1 Faculty of Dentistry, University of Oslo, 0455 Oslo, Norway; herlings7b@msn.com; 2 Informetrics Research Group, Ton Duc Thang University, Ho Chi Minh City 7000, Vietnam; 3 Faculty of Applied Sciences, Ton Duc Thang University, Ho Chi Minh City 7000, Vietnam; 4 Prosthodontic and Dental Implantology Department, College of Dentistry, King Faisal University, Al-Hofuf, Al-Ahsa 31982, Saudi Arabia; drzohaibkhurshid@gmail.com; 5 Department of Restorative Dentistry, College of Dentistry, Taibah University, Al Madinah, Al Munawwarah 41311, Saudi Arabia; mzafar@taibahu.edu.sa; 6 Islamic International Dental College, Riphah International University Islamabad 44000, Pakistan

**Keywords:** bioactive glass, dentistry, tissue regeneration, antimicrobial

## Abstract

At present, researchers in the field of biomaterials are focusing on the oral hard and soft tissue engineering with bioactive ingredients by activating body immune cells or different proteins of the body. By doing this natural ground substance, tissue component and long-lasting tissues grow. One of the current biomaterials is known as bioactive glass (BAG). The bioactive properties make BAG applicable to several clinical applications involving the regeneration of hard tissues in medicine and dentistry. In dentistry, its uses include dental restorative materials, mineralizing agents, as a coating material for dental implants, pulp capping, root canal treatment, and air-abrasion, and in medicine it has its applications from orthopedics to soft-tissue restoration. This review aims to provide an overview of promising and current uses of bioactive glasses in dentistry.

## 1. Introduction

At present, researchers in the field of biomaterials are focusing on tissue engineering and tissue regeneration [1]. In dentistry, a tissue engineering concept is not as new as we think; it is already developed with success of oral tissue regeneration such as in regard to dentine, pulp tissue scaffolds templates, periodontal membranes, and bone cements [2,3,4]. One of the accessible materials is bioactive glass (BAG). Larry L. Hench intended to develop a graft material compatible for the human body when he knew about the host rejection of inert metal and plastic materials used mainly for amputation cases [5]. This material turned out to be a glass that precipitated hydroxyapatite in aqueous solutions, with the ability of bonding to hard and soft tissues without rejection. The bioactive properties of BAG have caused a revolution in healthcare and apply to several clinical applications involving the regeneration of hard tissues in medicine and dentistry [5,6]. The application of nanotechnology help to synthesize BAG in the nano scale, this aids in coating the dental implant surfaces, orthopedic, and spinal implants [7,8]. Until now, over 1.5 million patients have been treated using Bioglass^®^ 45S5 worldwide [9].

## 2. Compositions of Bioactive Glass

Originally, BAG was commercially trademarked as Bioglass^®^ 45S5 composed of 45% SiO_2_, 24.5% Na_2_O, 24.5% CaO, and 6% P_2_O_5_ [10]. Class A BAGs mainly comprised of 40–52% SiO_2_, 10–50% CaO, and 10–35% Na_2_O. In addition, the glass composition may contain 2–8% P_2_O_5_, 0–25% CaF_2_, or 0–10% B_2_O_3_. Glasses of class B are usually bioinert with a silica content of > 60 weight % [11]. Besides, BAG may also consist of known biocompatible and bioactive minerals, including fluorapatite (FAP), wollastonite, diopside, and tricalcium phosphate [12,13]. The alkali-free (especially Na-free) BAG with the composition of 70% diopside, 10% fluorapatite, and 20% tricalcium phosphate is commercially known as FastOs^®^BG. Network modifiers such as CaO, Na_2_O, and P_2_O_5_ can be incorporated into the elemental Na_2_O-CaO-SiO_2_ composition to make the surface and silica network more reactive [14]. 

Na has been considered an essential component for the bioactivity, as it effectively disrupts the glass network. However, sodium-free BAG has been fabricated and shown to possess equal dissolution and bioactivity as traditional sodium contained BAG, thus discrediting Na as an essential component [15]. Further, it has been established that the rate of degradation and apatite formation is highly influenced by the connectivity of the glass silica network and the amount of phosphate. The presence of phosphate, or P_2_O_5_, was earlier assumed to be necessary for bioactivity. However, bioactive phosphate-free glasses have disproved this assumption [16]. Both CaO and Na_2_O can be replaced, respectively, by MgO and K_2_O. The apatite formation is promoted by the presence of MgO. Al_2_O_3_ and B_2_O_3_ can be added to influence the surface reaction and melting properties [17]. Furthermore, ions of Si, P, Sr, Cu, Ag, Zn, and F may be added to modify bioactivity and antimicrobial properties. Improved angiogenesis with Co has been shown when implanted in bone [18]. Improved antimicrobial properties may be achieved by an Ag [19], however, high concentrations have been reported to be cytotoxic [20]. Zn possesses antimicrobial properties as well. Additionally, alkali-free BAG doped with Zn showed improved apatite formation [21]. Cu, Mg, and Sr enhance the bioactivity of the BAG. Fluoride is particularly relevant in improving the bioactivity of dental applications by the formation of the more acid-resistant fluorapatite, rather than hydroxyapatite [22], and fluoride conjugated with BAG may enhance the remineralization of dentin and decrease the risk of dentin-matrix degradation [23].

## 3. Preparations of Bioactive Glass

Traditionally, glasses have been prepared by melt quenching, including Bioglass^®^ 45S5 [24]. During the process of melt quenching, ingredients in the form of powder are melted at high temperature, commonly above 1300 °C, and rapidly quenched for the atomic structure to freeze. However, this technique has flaws such as reduced bioactivity at higher sintering temperatures and the inability to fabricate porous scaffolds [25]. Commonly, heat treatment is used to relieve the glass of thermomechanical stresses due to rapid cooling. However, heat treatment may, at specific temperature ranges, result in the formation of different crystalline phases which may negatively affect the elastic modulus and strength, predisposing for mechanical failure, as is the case with thermal treatment of Bioglass^®^ 45S5 [26]. Heat treatment of silicate-based BAG results in the release of stresses from the glass as there is a possibility of formation of the crystalline phases along with the residual glassy phases which might affect the mechanical properties [26]. Furthermore, the glass particles can be sintered into glass-ceramic scaffolds, the crystallization, however, reduces the ion dissolution and the bioactivity.

**Figure 1:**
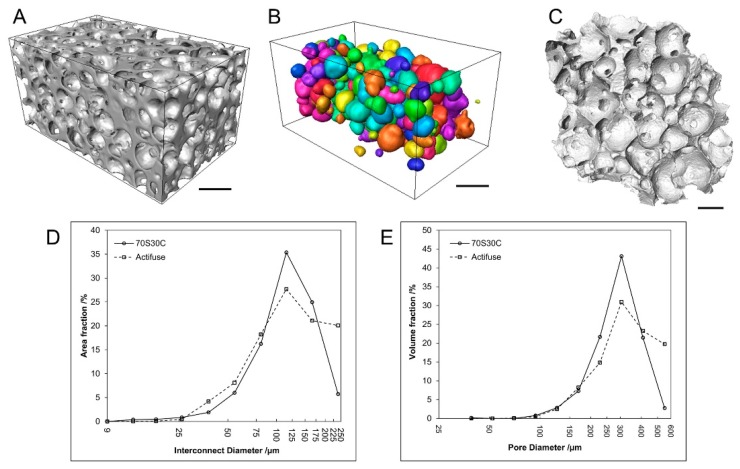
Characterization of bioactive glass scaffolds 70S30C: (**A**) Child volume reconstruction in the foam in micro-computed tomography, (**B**) separated pores from a child volume of a piece, (**C**) distribution of interconnecting sizes, (**D**) interconnecting sizes displayed as area fraction, and (**E**) pore size distributions of 70S30C. Scale bar = 400 μm. Reproduced from [28] with permission from Creative Commons Attribution License (CC BY 4.0) from Frontiers Media S.A.

From the early 1970s, the sol-gel technique rose up as an alternative method of glass synthesis [27], making it possible to produce a wide variety of glass compositions and shapes, such as fibers, coatings, scaffolds, and nanoparticles [1]. Midha et al. [28] produced bioactive glass scaffolds (70S30C, 70% SiO_2_, and 30% CaO) by a sol-gel foaming process thought to be suitable matrices for bone tissue regeneration (Figure 1). This technique uses precursors subjected to a variety of processes involving hydrolysis and condensation reactions, followed by low-temperature heat treatments. Sol-gel glasses possess higher porosity, apatite-forming ability, and increased surface area compared to the melt-quenched glasses, which have the advantage of higher mechanical properties [29].

## 4. Properties of Bioactive Glass

A bioactive material can interact with the biological environment to elicit a specific biological response, such as the formation of a hydroxyapatite layer with a bond forming between the tissue and material. Bone and teeth, enamel and dentin, consist mainly of mineralized hard tissue in the form of hydroxyapatite, a crystalline calcium phosphate, Ca_10_(PO_4_)_6_(OH)_2_ [30]. In contrast, bioinert materials do not elicit any specific responses or interact with the biological environment. However, they can result in a foreign-body reaction and the formation of a fibrous capsule. The fibrous capsule may result in micromovements and eventual failure of a prosthesis. Bioactive materials may be osteoconductive or osteoinductive [31].

The most bioactive glass has a superior surface area with a higher dissolution rate and thus faster apatite formation [32]. In addition, they have shown to increase the mechanical properties of such composite for natural bones and provide biomimetic nano-structuration enhancing cell adhesion.

The bioactive properties are influenced by the structure and composition of the glass, manufacturing techniques, and the rate of ionic dissolution. This is clearly illustrated when comparing BAGs to the traditional Bioglass^®^ 45S5. Bioglass^®^ 45S5 possesses several shortcomings which include the possibility of gap formation between the material and host tissues due to a rapid degradation rate [33,34]. The lack of porosity should not only be assigned to the composition but also to the applied process and the degree of particle aggregation [35,36]. Also, a Bioglass^®^ 45S5 may induce cytotoxic effects due to a high rise in pH due to high Na^+^ and Ca^2+^ leakage; this may additionally cause delayed hydroxyapatite formation [35,36,37]. The glass composition may not be favorable for the fabrication of porous scaffolds due to poor mechanical properties, such as too fragile [38,39]. Future research needs to improve the mechanical properties of the BAG.

### 4.1. Bioactivity of Bioactive Glass

Glasses are amorphous solids with the irregular organization of atoms, optically transparent, and brittle consisting of silica networks and are, therefore, often termed supercooled liquids [40]. Conventional glasses and BAGs differ in one aspect, their rate of dissolution. Conventional glasses, in general, are expected to have high durability and thus low dissolution rates. BAGs require specific dissolution rates for bioactivity. This is achieved by the addition of network modifiers, such as CaO and Na_2_O, to make the surface and silica network more reactive [16]. From glass dissolution to the formation of hydroxyapatite, bioactivity involves several steps.

Once in contact with body fluids (BF) or simulated body fluid (SBF), BAGs immediately undergo ionic dissolution and glass degradation via the exchange of H^+^ ions in the solution and Na^+^ and Ca^2+^ from the glass network. The ion exchange results in the formation of silanol groups (Si–O–H) due to the hydrolysis of the silica groups. An increased alkaline local environment develops due to the increase in OH^-^ concentration. The silica network is further degraded as the pH rises, forming orthosilicic acid and Si(OH)_4_ on the surface in the form of a negatively charged gel. The gel layer functions as a matrix for hydroxyapatite with precipitation sites [41]. Beneath the gel layer is a depleted alkaline surface layer on top of the bulk glass. On top of the gel layer, a layer of amorphous calcium phosphate forms. Precipitation and further mineralization occur due to the incorporated carbonate ions from the now supersaturated solution, thus the concentration of Ca- and Si-ions in solution are critical; about 88–100 ppm and 17–20 ppm of the respective ions are required. The newly formed hydroxyapatite enables growth factors to adsorb to the surface, as well as attachment, proliferation, and differentiation of osteoprogenitor cells by cytokines and extracellular matrix components expressed by the upregulation of several genes [41]. Although the tissue bonding properties of the BAG are still not precise, collagen and glycoproteins are believed to incorporate the surrounding bone tissue into the hydroxyapatite layer. As the hydroxyapatite grows inwards, the BAG starts to resorb and gets replaced by growing bone tissue [42]. The glass particles usually have a size of 90–170 μm, which affects their resorption rate. Particle sizes < 150 μm readily degrade as orthosilicic acid is released during the formation of the gel layer. Osteoclasts, once incorporated in the growing bone, break down larger particles [43] resulting in a more extended period of resorption and stronger bone [44].

### 4.2. Antimicrobial Properties

Dental implants or prosthetic joints are surgically inserted to replace lost tissue and increase the function and quality of life of the patients [45]. However, implants carry a risk of developing infections such as peri-implantitis (PI) or periprosthetic joint diseases (PJI). These infections result in increased morbidity and mortality, as well as resorption of surrounding bone tissue and eventual loosening of the implant. Tomasi and Derks [46] estimated a weighted mean prevalence of 22% for PI. Any artificial joint may develop PJI. PJI occurs in 0.2–9% of prostheses and is one of the most frequent indications of revision and replacement of the joint prosthesis making up for 15% of hip prostheses and 25% of knee prostheses [47]. Infection occurs due to the establishment of bacterial biofilm on the surface of the implant. Biofilm is a layer of microbial communities adhering to a surface via a robust polysaccharide matrix and is known to be about a thousand times more resistant to antibiotic (AB) therapy compared to planktonic bacteria. Bone infections pose an additional challenge of the reduced local effect of antibiotic treatment owing to insufficient vasculature [48] or areas of devitalized bone [49]. Additionally, the increasing prevalence of antibiotic-resistant bacteria, including multidrug-resistant bacteria, results in the ineffective treatment of bacterial infections with antibiotic therapy, including AB-loaded bone substitutes as carriers [50].

Surgical debridement or osseous resection followed by placement of a bone substitute in the defect has shown positive treatment outcomes for established PI cases. PJI can be managed by surgical debridement and AB therapy. However, for resistant pathogens or loosened prosthesis, revision is necessary, which results in reduced quality of life [51].

**Figure 2:**
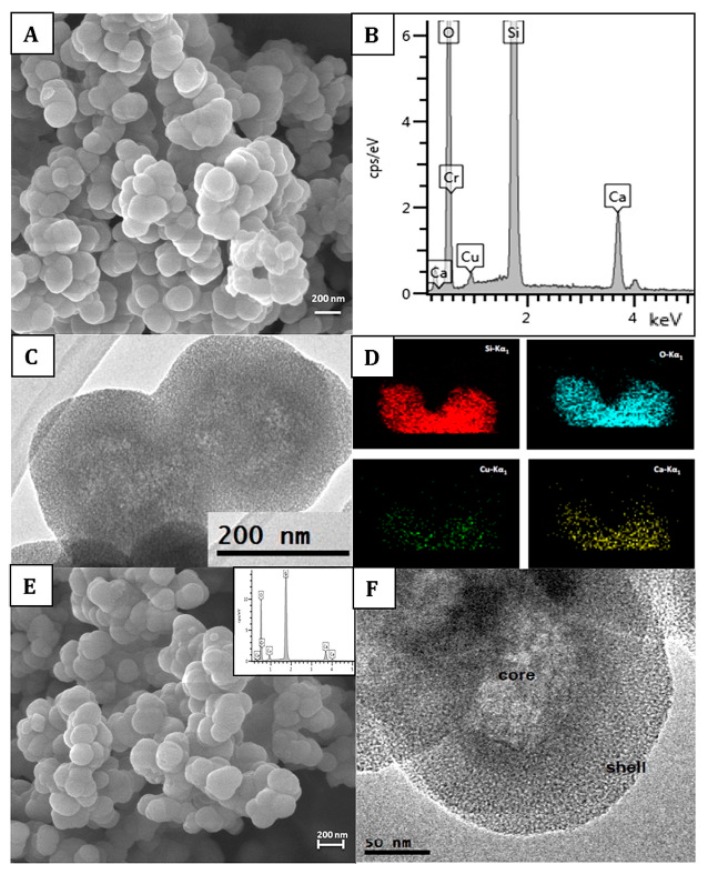
Characterization of Cu-doped mesoporous SiO_2_-CaO glass (Cu-MBG) 2%: SEM image (**A**), EDS spectrum (**B**), TEM image (**C**), and EDS mapping showing uniformly distributed in the nanoparticles of Si (red), O (blue), Cu (green), and Ca (yellow) (**D**). Characterization of Cu-MBG 5%: SEM image (**E**), related EDS spectrum (inset), and TEM image (**F**). Reproduced from [56] with permission from Elsevier.

BAGs, specifically BAG-S53P4, have shown to possess broad-spectrum antimicrobial properties with no observed resistance to date [52]. *S. aureus* is among the most common bacterial strains implicated in PJIs and a major biofilm contributor. However, S53P4 has proved to reduce the biofilm mass in vitro conditions [53,54]. The antibiofilm activity has further been observed to affect several multi drug resistant strains isolated from PJIs [53]. Additionally, an increased antibiofilm effect is observed with the incorporation of antimicrobial molecules into the BAG [55,56,57]. S53P4 has successfully been used in the treatment of osteomyelitis [58]. The antimicrobial and antibiofilm effects of bioactive glasses differ from that of conventional Abs, as once embedded in the body, bioactive glasses increase the pH and osmolarity locally, which creates an environment unfriendly for bacterial growth and adhesion [55,59]. Bari et al. [56] developed Cu-doped mesoporous SiO_2_-CaO glass (Cu-MBG) by an ultrasound assisted one-pot synthesis (Figure 2). Cu-MBG nanoparticles showed antibacterial effects against 3 bacterial strains (*E. coli*, *S. aureus*, and *S. epidermidis*). The Cu-MBG can be a promising and versatile platform for bone and soft tissue regeneration.

As alkalinity is considered the primary antimicrobial mechanism, Bioglass^®^ 45S5 is considered more effective. However, S53P4 presents a more delicate balance between antimicrobial properties, alkalinity with a pH of 7.9, and osteogenicity [60]. Particle size influences the antimicrobial properties as well; small particle sizes increase the surface area and the antimicrobial effect [54,61]. Alkali-free BAG doped with ZnO and SrO synthesized by melt quenching exhibited antimicrobial properties against strains of *Staphylococcus aureus* and *Escherichia coli*. The results suggest that BAGs can still provide antimicrobial properties in the absence of alkalinity [21]. These properties make BAG perhaps the ideal bone substitute in the treatment of bone infections such as osteomyelitis and peri-implant infections [62,63]. The US Food and Drug Administration (FDA) has approved Bioglass^®^ 45S5 and S53P4 for clinical applications where antimicrobial properties are desired [64].

BAG also exhibits antimicrobial properties against pathogens associated with sinusitis, which makes it excellent for sinus augmentation and repairing of the orbital floor defects. A communication often exists between these anatomical structures, and infection from maxillary sinus can quickly spread to an orbital floor implant, necessitating implant removal [65]. Hence, BAG S53P4 can be used for these applications due to slow resorption and antimicrobial effect.

BAG-coated dental implants have shown promising results with reduced bone loss in experimentally induced PI in beagle dogs [66]. A recent in vitro study showed reduced biofilm formation of putative periodontal pathogen strains, in addition to *S. mutans* [67]. Besides, reduced growth of periodontitis-associated and cariogenic bacteria, as well as *Enterococcus* facials, has been reported using BAG containing propolis, a naturally occurring compound in beehives [68]. Additionally, BAGs can incorporate hydrophilic as well as hydrophobic compounds into their structure suggesting several undiscovered combinations of compounds may be achievable to increase antimicrobial efficiency [55], strengthening a future antimicrobial role of bioactive glass in dental applications. Another assessment was done on machined Ti6Al4V threaded dental implant coated with hydroxyapatite and bioactive glasses in human jaws, the outcome is very promising and futuristic. The observed BAGs are safe and effective like hydroxyapatite for enhancing osseointegration [69].

## 5. Clinical Applications of Bioactive Glasses in Dentistry

**Figure 3:**
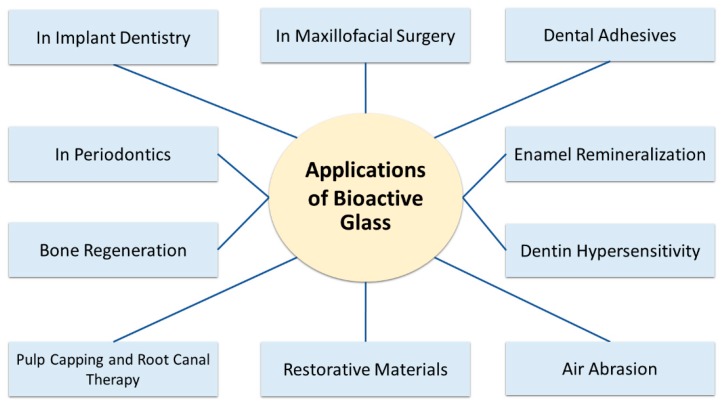
Various clinical applications of bioactive glass in dentistry.

The compositional similarity to the bone and tooth structure combined with the bioactive properties and apparent antimicrobial properties inspired the research of BAGs in clinical application in dentistry and were first used as bone substitutes in dentoalveolar and maxillofacial reconstruction, periodontal regeneration, and implants [36,70,71]. Various applications have been reported in the last two decades, illustrated in Figure 3.

### 5.1. Dental Adhesives

Dental adhesives make it possible to achieve adhesion, or bonding, of a compound or material, such as dental composite or orthodontic brackets, to natural tooth tissue. Bonding of dental resin composite to tooth overcomes the challenge of adhering hydrophobic resin composite to the hydrophilic tooth surface. The adhesive, therefore, functions as the link between the two substances. The adhesion of orthodontic brackets leads to favorable conditions for bacterial colonization which may result in demineralization and white spot lesions (WSLs) [72]. Prevention of WSLs involves regular tooth brushing and fluoride dentifrices, mouthwash, or varnishes. This requires a high degree of patient compliance and additional costs. Researchers aiming at the prevention of WSLs have focused on fluoride-releasing sealants, primers, and adhesives to achieve continuous fluoride release throughout orthodontic treatment. However, fluoride addition compromised the mechanical properties of the resin-based adhesives and fluoride release depleted over time. 

The tooth preparation for composite restoration produces a smear layer, chiefly containing tooth substance and bacterial remnants that cover the surface and occlude the dentinal tubules. To remove the smear layer and to expose the dentinal tubules and the collagen network for better infiltration of the bonding resin components, acid-etching is done. However, the low pH of the etchant may induce the activation of matrix metalloproteinases (MMPs), which degrade the collagen network of dentine. Poorly infiltrated resin interfaces in etched dentine thus may result in the degradation of the hybrid interface layer, decreased bond strength with increased risk of material degradation, and bond failure [73,74,75,76].

The effects of two experimental resin bonding systems containing micro-fillers of Bioglass^®^ 45S5 or Zn-polycarboxylated BAG were evaluated on the resin-bonded dentine interface after storage in SBF for three months. The high content of zinc may have protected the collagen network from the action of MMPs in addition to the pH rising property of BAG [77]. Compared to the BAG-free bonding system, the BAG-containing bonding systems reduced micro-permeability by remineralization of mineral-deficient areas as well as showing an increase in modulus of elasticity and hardness along with the dentine interface both after 24 hours and three months of SBF immersion [78].

A crystallized bioactive glass-ceramic, Biosilicate^®^, (Na_2_O 23.75 weight %, CaO 23.75 weight %, SiO_2_ 48.5 weight %, and P_2_O_5_ 4.0 weight %) was recently released [79]. It showed promising clinical results for bone grafting, and in combination with titanium implants. It has also been advocated as an alternative treatment of dentin hypersensitivity and for the total-etch adhesive bonding system [80]. Biosilicate^®^ particles, when in contact with dentin, react with the tissue inside the dentinal tubules resulting in dentinal occlusion by hydroxyapatite and, therefore, provide a stronger bond [79]. A suspension of Biosilicate^®^ has been shown to increase the bond strength of adhesive systems in both mineralized and demineralized dentin when applied before application [81].

The incorporation of fillers of niobophosphate BAG into a commercial adhesive produced higher microhardness and radiopacity compared with the adhesive without BAG. The mechanical properties were not compromised. Additionally, apatite formation was noted [82]. A novel BAG-resin orthodontic adhesive containing fluoride appeared to promote apatite formation in neutral and acidic conditions and may have a clinical role in remineralization and prevention of WSLs around orthodontic brackets.

Commercial orthodontic bonding agents were mixed with BAG, Ag- or Zn-doped, using flowable resin. The addition of BAG produced a demineralization-free zone up to 200 to 300 µm away from the bracket after pH cycling. In comparison, all surfaces not covered by BAG-free bonding agents, used as controls, were demineralized. Additionally, the experimental bonding agents exhibited significant inhibition of *S. mutans* compared to the controls [83]. These results suggest their possible use in orthodontic practices.

### 5.2. Enamel Remineralization

Early caries lesions that have yet to cavitate, such as WSLs, may be arrested and remineralized with regular plaque removal and fluoride; operative treatment may then be avoided. Fluoride is widely used in toothpaste, varnishes, and mouth rinse to control caries and promote remineralization. An alternative to fluoride is the milk protein-derived casein phosphopeptide-amorphous calcium phosphate (CPP-ACP), commercially known as Recaldent™ [84]. A recent randomized clinical trial did find comparable results in terms of remineralization of WSLs of CPP-ACP and fluoride gel in children. However, the best WSL remineralization was achieved using a combination of CPP-ACP and fluoride [84]. Conversely, WSLs can be remineralized by the application of BAG [85]. Bioglass^®^ 45S5 has been extensively studied regarding the remineralization of WSLs. Taha et al. [86] evaluated the effectiveness of bioactive glasses in inducing remineralization compared to topical fluoride and CPP-ACP treatment. They concluded that bioactive glasses may enhance enamel remineralization more effectively and earlier. However, clinical research is lacking.

Novamin^®^ has an identical composition to Bioglass^®^ 45S5, but with an average particle size of 18 µm, and is used as the active ingredient in the commercial toothpaste, Sensodyne^®^ (GlaxoSmithKline), for remineralization and reducing hypersensitivity [87]. Novamin^®^ is a calcium–sodium–phosphate silicate glass that releases calcium and phosphate ions. These ions increase the pH and result in precipitation of calcium phosphate and mineralization into hydroxyapatite as conventional BAG [88]. While CPP-ACP or other calcium-based products provide an initial calcium burst, Novamin^®^ exhibits a continuous calcium release [89]. However, the availability of only in vitro and in situ studies and lack of randomized clinical trials (RCTs) precludes the clinical use of Novamin^®^ for enamel remineralization [90]. Additionally, fluoride-doped BAG exhibited potential for the use in dental applications, such as dentifrices and restorative materials. BAGs doped with 2.5%, 5%, and 7.5% mol fluoride showed stable daily fluoride release above 1.2 ppm over an observational period of 6 months [91]. A BAG with the combination of fluoride and high phosphate content, commercially known as BiominF^®^, resulted in the formation of FAP rather than fluorite, CaF_2_. The high phosphate content serves as a source of delivery of all the necessary ions of FAP, Ca_5_(PO_4_)_3_F [92]. The remineralization efficacy of BiominF^®^ was compared to a BAG-containing dentifrice and Novamin^®^ in vitro using micro-CT. BiominF^®^ showed better remineralization at 5 min and 24 h [93]. More in vivo studies are required to justify the clinical effectiveness of BiominF^®^.

### 5.3. Dentin Hypersensitivity

Dentin hypersensitivity (DH) is characterized as sharp and short-lasting dental pain to a tactile, chemical, osmotic, evaporative, or thermal stimulus. DH may be elicited by exposed dentin due to erosion, attrition, abfraction, abrasion, gingival recession, or periodontal disease. The most accepted theory on DH is the hydrodynamic theory in which stimuli induce fluid movement in the dentinal tubules causing mechanoreceptors close to the pulp to excite the nerve terminals of Aδ fibers resulting in the perception of the characteristic pain [94,95]. DH, based on the hydrodynamic theory, can either be managed by blocking nerve excitation or by sealing the dentinal tubules. Excitation is proposed to be blocked by raising the extracellular concentration of potassium ions around the nerve fibers, which blocks repolarization thereby preventing the generation of the action potential [96]. Occlusion of open dentinal tubules reduces the dentinal fluid flow [97].

The over-the-counter products used in the conservative management of DH include glass ionomer cement (GIC), bonding agents, and dentifrices [98]. Glass particles may be combined with these products. BAG formulations provide therapeutic relief via occluding the dentinal tubules by binding to collagen fibers and depositing hydroxyapatite [99]. Novamin^®^, with a particle size of 18 µm, was introduced in 2004 as an ingredient in toothpaste, Sensodyne^®^, to treat DH [100]. Sensodyne^®^ has been widely recommended by dentists [98]. PerioGlas^®^ as well has been successful in treating DH. A firm surface affinity of these two formulations to collagen eases dentin bonding, thereby occluding the tubules [101]. Increased amounts of BAG correlate to increased tubule occlusion. BAG applied to dentin discs alone can be easily displaced by rinsing. The substitution of BAG for silica in toothpaste provided resistance against pH rinse and brushing off the occluded tubules [89,102]. BAG particles synthesized by the sol-gel technique provided an increased surface area and rapid bonding to dentin compared to the melt-quenched.

Although there are numerous in vitro shreds of evidence, limited in vivo data exist for the clinical effectiveness of Novamin^®^ to treat DH. Gendreau et al. reviewed the available clinical studies and supported the clinical effectiveness of Novamin^®^ in toothpaste [103]. When compared to a potassium nitrate (5% KNO_3_) containing dentifrice formulation, a 5% Novamin^®^ formulation had significantly lower pain scores and a longer duration of relief [104]. Toothpaste with fluoride-containing BAGs that form FAP is proposed to provide a more effective treatment of DH [92]. FAP-forming BioMin-F^®^ has been compared in an RCT for its clinical desensitizing property to NovaMin^®^ and a standard fluoride dentifrice, all in 5% formulations. All groups exhibited significantly decreased visual analog scale score for individual and thermal sensitivity after 60 days. However, BioMin-F^®^ was more effective both immediately and in the long term [104]. The crystallized BAG, Biosilicate^®^, regarded as an alternative for the treatment of DH, was evaluated in a long-term clinical study [79]. A dispersion of Biosilicate^®^ in distilled water proved to be effective in treating DH and provided relief for a follow-up period of 6 months [105].

### 5.4. Air Abrasion

Novamin^®^, or BAG in general, exhibits a hardness (Moh) of 7 GPa, which is higher than that of enamel (3.5 GPa) [106]. More rounded glass particles are less abrasive. With the increase in particle size, the abrasiveness increases. The cementoenamel junction is prone to DH due to the wear of enamel. Therefore, the use of less abrasive dentifrices is advised on the outer enamel layer that is susceptible to wear. The addition of fluoride or strontium reduces the hardness of BAG and should be included in dentifrices. In vivo studies evaluating the abrasiveness based on ionic compositions of different BAG dentifrices is therefore required. Novamin^®^ has been used for teeth whitening owing to its abrasive properties. Surface stains can be removed by the high-pressure airflow of ceramic particles. Patients had subjective reductions of DH and whiter teeth using airflow with Novamin^®^ particles compared to conventional sodium bicarbonate particles [107].

The most significant enamel damage due to orthodontic treatment occurs when the residual orthodontic adhesive is removed on completion of the procedure. Tungsten carbide at slow speed has been conventionally used for the purpose. Particles of alumina and BAG 45S5 have been studied in vitro to assess the enamel damage during air abrasion and compared to tungsten carbide bur for removal of residual orthodontic adhesive. BAG 45S5 yielded the least enamel damage, followed by alumina and tungsten carbide. As revealed by the scanning electron microscope (SEM) images, QMAT3, a BAG with hardness lower than that of enamel showed minimal enamel injury compared to BAG 45S5 (Sylc™) and tungsten carbide bur [108]. QMAT3, thus, seems to provide a conservative approach for removal of orthodontic adhesive.

### 5.5. Restorative Materials

The restorative materials currently available can mimic the tooth in appearance, form, and function, but lack bioactive properties. During a cavity restoration, glass ionomer cement or resin composite undergo some degree of polymerization shrinkage [109]. A microgap thus formed may widen due to discrepancies in the mechanical properties of the tooth and the restorative material. The gap often inaccessible to routine dental hygiene techniques creates a favorable milieu for bacterial growth resulting in secondary caries, the most common reason for the failure of dental restorations [110]. Additionally, the tissue-saving approach during the removal of caries may leave residual bacteria in affected tissue [111]. The development of dental restorative materials able to remineralize or repair demineralized dentin, following the bacterial invasion, has been one of the areas of dental biomaterial research. The longevity of dental restorations can be achieved by creating a tight bond to the tooth and a hostile environment for bacteria. Bonding agents with bioactive properties may provide a sealed interface by hydroxyapatite precipitation [112]. Bioglass^®^ 45S5 has shown to induce dentin remineralization.

BAG was first incorporated into a resin composite in the non-silanated format 5, 10, and 15 weight % with a filler content of 72%. Its mechanical properties were higher than the control, 0 weight % BAG, after two months of exposure to bacterial challenge and aqueous media [113]. The BAG composites showed cytotoxicity due to the release of unreacted monomers compared to commercially available resin composites [114]. Flowable resin composite materials proved to inhibit the growth of oral microbes including *E. coli* and *S. mutans* without compromising the bond strength [115].

Resin composites with BAG and fluoride enhanced dentin remineralization and eliminated enzymatic degradation at the dentin interface. The remineralizing capacities of F-BAG and BAG resin composites were compared in the samples stored for 3 and 30 days in artificial saliva. F-BAG not only exhibited the greatest remineralization of dentin but also reduced the enzyme-mediated degradation of the dentin collagen network. This suggests the benefits of incorporating F-BAG into resin composites over conventional BAG, 45S5 [116]. Ag-doped BAG resin composite was investigated for its antibacterial properties and bioactivity. The increased concentration of Ag-BAG resin composite increased the number of dead bacteria in biofilm and apatite formation when compared to control samples (BAG-free resin composite). Mechanical properties showed no significant differences compared to control samples. The findings suggest that Ag-BAG resin composites may be instrumental in inhibiting secondary caries formation [117].

Mechanical properties vary between BAG resin composites. Experimental resin composites in which 0–15 weight % of the fillers were replaced by ground BAG were stable when immersed in brain heart infusion media for two months and exhibited similar mechanical properties except for decreased fracture toughness and fatigue resistance when compared to three commercial composites [113]. Dentin bond strength was investigated in a resin composite with a varying amount (0–40 weight %) of BAG and 70% filler content after artificial aging in water. As the weight % of BAG increased, there was a linear decline in the bond strength of BAG resin composite [118]. Similar experimental BAG resin composites were investigated for their flexural strength, flexural modulus, modulus of resilience, and material reliability after artificial aging in water and ethanol. Flexural strength and modulus decreased linearly as BAG content increased and were further degraded by the artificial aging. As per ISO 4049, minimum flexural strength was achieved up to 20 weight % of BAG. Additionally, modulus of resilience and degree of conversion were decreased with BAG incorporation [119]. Experimental pit and fissure sealant (0–50 weight % BAG) exhibited a dose-dependent decline in flexural strength and an increase in water sorption with increasing content of BAG [120]. However, experimental composites with 0–15 weight % of sodium-free BAG and reinforcing fillers up to 72 weight % showed similar flexural strength as BAG-free resin composites [113].

Resin composites with BAG filler particles exhibit antimicrobial and bioactive characteristics, which are instrumental in the prevention of secondary caries. However, their mechanical, optical, or adhesive properties may be compromised [113]. Silanization of filler particles is used in conventional resin composites for improved mechanical properties. However, it reduces ion release and thus impairs bioactivity [121,122]. Resin hydrophilicity may be decreased to improve aging resistance of a resin composite. However, this will also reduce the remineralizing properties of the composite. The heterogenic results reflect the notion that mechanical properties and degradation are variable regarding BAG resin composites.

Glass ionomer cement (GIC) primarily consists of fluoride–aluminosilicate glass and polyacrylic acid and may be modified by adding methacrylate resin monomers (resin-modified GIC, rmGIC), for better mechanical properties, stronger adhesion, and lower solubility. GIC is known for its fluoride release, remineralizing properties, and direct chemical bonding to the tooth. BAG particles have been incorporated into formulations of GIC to regulate remineralization [123,124]. A GIC based on BAG and polyacrylic acid showed the similar acid-base reaction for setting between glass particles and polyacrylic acid as that of the conventional GIC [125]. It is suggested that the iron-rich matrix formed during the setting of GIC forms an osmotic gradient that allows water to be absorbed by the matrix. This water absorption creates an aqueous environment for BAG particles to react.

Additionally, increased water absorption is reported with rmGIC, which justifies greater bioactivity of BAG-rmGIC than GIC [126]. Besides, BAG-rmGIC has superior remineralizing properties to that of rmGIC as shown in an in vitro study where the flexural strength of demineralized dentin immersed in SBF containing BAG-rmGIC was significantly higher than in SBF alone or SBF with rmGIC [126]. An in vivo study in intact beagle dog teeth involved class III restorations with 10–30 weight % BAG-rmGIC, BAG-GIC, and BAG-free GIC as control. Restorations were followed for 1, 3, and 6 weeks. A uniform layer of calcium phosphate formed on the surface of BAG-rmGIC and mineral depositions were noted at the dentin-restoration interface. Similar depositions occurred in deeper parts of the dentinal tubules. From these findings, BAG-rmGIC appeared promising for remineralization [127]. The antimicrobial properties of BAG-GICs with 10–30 weight % of BAG (S53P) against *Streptococcus mutans* and *Candida albicans* were assessed in vitro. Antimicrobial properties against *S. mutans* were exerted by BAG-GICs at 30 weight % BAG, while BAG alone exerted antimicrobial effects against both *S. mutans* and *C. albicans* [128]. These findings strengthen the potential clinical role of BAG-GICs in preventing secondary caries. The rmGIC with low amounts of nanoparticle BAG (nBAG) increased the flexural strength of the material, while high concentrations exhibited detrimental effects on the mechanical properties due to reduced bonding between the glass particles and the resin matrix [129]. Despite the bioactive and antimicrobial properties exerted by the incorporation of BAG particles to GICs, the mechanical properties were compromised. This may restrict the potential clinical uses of BAG-rmGIC to areas of low mechanical stresses and in need of bioactivity such as root surface fillings and liners [130,131].

### 5.6. Pulp Capping and Root Canal Therapy

The interest of BAG is also present in endodontic management [132,133,134]. For an exposed dental pulp indicated for partial pulpotomy or pulp capping, the choice of a pulp-capping material is important among other factors that determine the treatment success [132]. A pulp-capping material should be able to provide a tight seal, be biocompatible, antibacterial, and easy to handle. Additionally, it should promote the formation of a dentin bridge to protect the pulp. Although the dentin bridge formed by calcium hydroxide (CH) is incomplete due to tunnel-like defects, it has been used in several endodontic applications, such as pulp capping. Long setting time and delicate handling during application are other notable drawbacks of calcium hydroxide [133]. BAG has been investigated for pulp capping owing to its putative dentinogenesis property. An in-vitro study showed that the ions released by the sol-gel nanoporous BAG particles did not inhibit the growth of human dental pulp stem cells (hDPSCs) but showed a high density of mineralized nodules [134].

Sol-gel derived BAG when used for direct pulp capping stimulated the formation of a dense dentin bridge with inflammatory responses similar to mineral trioxide aggregate (MTA), as shown in mechanically exposed pulps of rats [135]. More unfavorable inflammatory responses have been observed using melt-derived BAG powders compared to that of sol-derived BAGs [136]. The extended setting time and undesired physical properties of MTA can be modified by the addition of BAG, as evident by a study using an MTA-like cement composite of wollastonite and BAG.

Once microorganisms have reached the pulp, root canal treatment is indicated. A sturdy and dimensionally stable root filling material that prevents bacterial leakage is necessary, in addition to a tight coronal seal [137,138]. BAGs have been implemented in endodontic root filling materials as well. The endodontic obturation thermoplastic polymer commercially known as Resilon™ [139] utilizes BAG as filler particles. Bio-Gutta, a gutta-percha (GP) mixed with Bioglass^®^ 45S5, has emerged as an alternative to classical GP as it can bond to dentin walls and does not require any sealers [140]. GP undergoes shrinkage during cooling, and difficulty to adapt to the canal morphology without heating makes it necessary to use a sealer to seal the gap. This technique, however, predisposes to microleakage through interfacial gaps due to varying binding strength to dentin and GP [137]. Bio-gutta is an obturating material with a high degree of biocompatibility [141] comparable to GP. Additionally, it provides a tight seal, increases the pH, and provides antimicrobial action. Bio-Gutta is based on the premise that the formation of calcium phosphate would precipitate on the material surface under moist conditions and provide self-adhesiveness and a tight seal [142,143]. Polyisoprene (PI) and polycaprolactone (PCL) were mixed with Bioglass^®^ 45S5 up to 30 weight % separately to develop root canal filling materials with high sealing ability making the need for a sealer obsolete. GP and Resilon™ served as controls. Both BAG+PCL and BAG+PI showed hydroxyapatite precipitation and improved immediate sealing ability with no observable leakage in vitro when compared to control samples [144]. Thus, Bio-Gutta, PCL, and PI with BAG may serve as clinical alternatives to conventional GP.

### 5.7. Bone Regeneration

With the increase in an aging population, difficult-to-heal bone defects and subsequently the need for synthetic bone graft substitutes are expected to increase. Bone defects may be caused by trauma, congenital or developmental disorders, deformities, cancer, sequelae of surgery, periodontitis, or osteomyelitis [145,146]. Bone defects result in socioeconomic burdens and a decrease in quality of life. Of approximately 2 million procedures requiring bone grafts performed annually worldwide, 700,000 involve cranial bone repairs [146]. The bone-grafting materials that are currently in practice include autologous bone grafts (BGs), allogenic BGs, xenografts, and synthetic BGs. The autologous BG is regarded as the gold standard as it combines all the necessary features for bone regeneration, carries no risk of adverse immune reactions, and is highly osteogenic [145,147]. Since it results in a second bone defect and donor site morbidity [148,149], the amount of graft harvested is thus limited. The qualities of an ideal grafting material set by Janicki and Schmidmaier are not fulfilled by autologous, allogenic BG, or xenografts [147]. To meet the increasing demand for BGs, the market is shifting towards synthetic BGs [150]. This adds an advantage to a synthetic BG as it can be tailor-made to possess the ideal qualities of a bone-grafting material.

**Figure 4:**
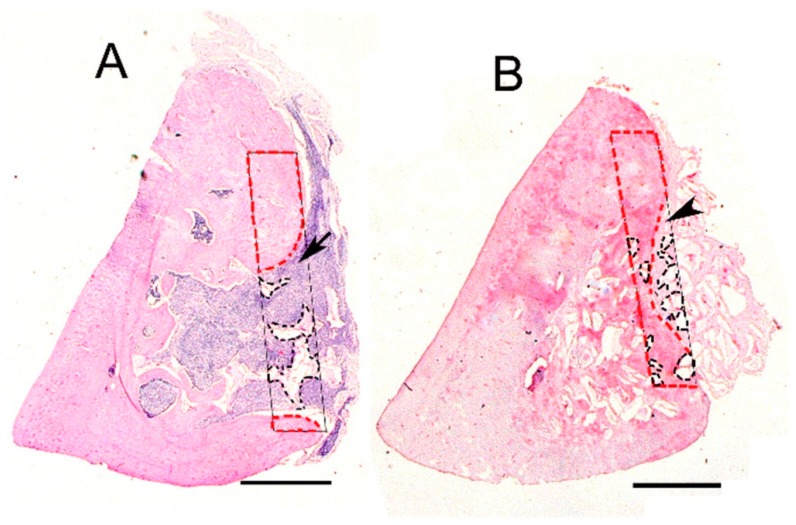
Hematoxylin and eosin stained transverse sections from a tibia implanted with dry 70S30C granules (**A**) or NovaBone^®^ (**B**). Bone (red lines), material (black lines), fibro-inflammatory tissue (arrow), fibrous tissue (arrowhead). Scale bar = 1.3 mm. Reproduced from [28] with permission from Creative Commons Attribution License (CC BY 4.0) from Frontiers Media S.A.

Midha et al. [28] found that bone growth of dry, wetted, and preconditioned 70S30C scaffolds were 10 ± 1%, 21 ± 2%, and 39 ± 4%, respectively, at 11 weeks (Figure 4). The preconditioned scaffolds degraded and were replaced with new bone. The composition of bioactive glass should be redesigned if sol-gel scaffolds are used without preconditioning to avoid excess calcium release.

### 5.8. In Periodontics

Periodontitis is a widely prevalent chronic inflammatory disorder of the periodontium characterized by the formation of deepened soft tissue pockets between gingiva and tooth roots, resorption of alveolar bone, loss of clinical attachment level, and subsequent loosening of teeth [151]. Periodontitis also increases the risk of peri-implantitis with the loosening of the dental implant as a consequence [152,153]. To improve the prognosis of teeth or dental implants, regeneration of osseous defects is necessary [154]. Tooth loss induces local resorption of the alveolar ridge. Enough height and bone volume of the alveolar ridge is necessary for the insertion of dental implants. As previously discussed, BAG is an excellent bone-graft material and has been widely used clinically as PerioGlas^®^ in the regeneration of periodontal bone defects. PerioGlas^®^ has an identical formulation to that of Bioglass^®^ 45S5 [71,155].

PerioGlas^®^, with particle size within 90–710 µm, can be pressed into bone defects, has been extensively used in periodontal surgical procedures to stimulate bone regeneration, especially in interproximal bone defects, and is beneficial due to its hemostatic effect on trabecular bone [71,156,157]. The formulation has also been evaluated radiographically in the treatment of apical osseous defects by endodontic surgery, which resulted in a higher success rate and earlier bone regeneration [158]. Another Bioglass^®^ 45S5 derived commercial BAG used in periodontal surgery is the ERMI^®^, Endosseous Ridge Maintenance Implant, released in 1988. ERMI^®^ is a prefabricated Bioglass^®^ cone that can be inserted into fresh extraction sockets. A study with a 5-year follow-up showed cone retention of 85.7% and proved to be safe for supporting dental structures and dentures [159].

PerioGlas^®^ and autogenic bone graft had comparable regenerative attachment gain in the treatment of grade II furcation involvement and intraosseous periodontal defects in RCTs [160,161]. A meta-study concluded that the treatment of intrabony defects with BAG yields a significant improvement in probing depth and clinical attachment level compared to both active controls and open flap debridement [162]. Additionally, BAGs have shown a better treatment approach than conventional methods, such as closed or open debridement [159]. However, true regeneration needs to be assessed histologically. True regeneration needs to demonstrate the formation of new functional periodontal ligament (PDL), alveolar bone, and cementum at the treated site [163]. This is not evident with the Bioglass^®^ 45S5 formulations as they only show bone formation, without cementum or PDL, and therefore provide repair rather than regeneration [164,165]. The granular nature of commercial BAGs in periodontal therapy, such as PerioGlas^®^, is unable to provide space and unable to support loading and may collapse during healing [156].

### 5.9. In Implant Dentistry

Dental implants (DI), also termed endosseous implants, are screw-shaped devices inserted in the alveolar bone to support prosthodontic constructions to improve function and appearance [166]. To achieve adequate retention in bone, osseointegration, direct contact between the implant surface and bone tissue is needed [167]. Titanium-based alloys are the most widely used materials for DIs. They are highly biocompatible and osteoconductive but are bioinert [37]. However, they provide attachment for the osteoprogenitor cells, osteoblasts, and are undesirable for microorganisms [168,169]. There are also reports of failed osseointegration of Titanium-DIs [170,171]. For DIs to be successfully integrated, they must be mechanically strong enough to withstand chewing forces over time, not induce inflammatory or foreign body tissue responses, and promote bone apposition in terms of osseointegration. To achieve stability and osseointegration, a healing period of 3-6 months is necessary to avoid early failure [172,173]. The bioinert nature of the Ti-DIs may benefit from the addition of BAG as BAG might help implants bond actively to the bone, and provide antimicrobial protection and a reduction in total treatment time [169,174,175]. Until today, no BAG coating for DIs is commercialized for clinical use.

A major challenge in the BAG coating of DIs is the thermal expansion coefficient (TEC) of BAG. During cooling, the glass and metal will shrink at different rates, which makes the coating prone to cracks. Ideally, a slightly lower glass TEC than the metal may prevent cracking [176]. Adjustment of glass TEC is achievable by increasing the amount of silica, or partial substitution of CaO by MgO and Na_2_O by K_2_O [177,178]. Several methods of surface deposition have been investigated in the pursuit of a reliable BAG coating of DIs, including glazing [177,179,180,181], sol-gel deposition [182,183], electrophoretic deposition [184,185], pulsed laser deposition [186,187], ion-beam [188], and radio-frequency magnetron sputtering [189,190,191]. The radio-frequency magnetron sputtering (RF-MS), which yields a coating with excellent adherence and purity even in complex geometrical objects, seems promising [192,193]. In vivo animal studies of BAG-coated Ti-DIs osseointegrated with significantly more surrounding bone tissue than control DIs [194,195]. Uniform and mechanically resistant BAG-coatings by RF-MS were assessed in the mandibular bone of dead pigs. Biocompatibility assays proved strong cellular adhesion and proliferation of dDPSCs [196], further emphasizing the role of BAG in the development of a new generation of dental implants.

### 5.10. In Maxillofacial Surgery

Bioglass^®^ 45S5 and formulations based on it have been heavily investigated in oral and maxillofacial surgery, OMFS. Compared to other calcium phosphate-based compounds such as hydroxyapatite and tricalcium phosphate employed in osseous repair, BAG induces bone formation at higher quantity and quality and a faster rate [197]. One of the commercial products used mainly for the repair of defects in maxillofacial applications is Biogran^®^, which differs from PerioGlas^®^ in its particle sizes (300–360 µm) [198]. NovaBone^®^, another Bioglass^®^ 45S5-based formulation, can be mixed with blood from the defect to form a putty to fill the site [10]. Large defects such as mandibular advancements, or mastoid or orbital floor fractures, can be repaired using BonAlive^®^, an S53P4 particulate with a mean particle size of 1–4 mm [43,199]. Large bone defects can also be treated using granular BAG mixed with autogenous bone in small amounts with high success and a considerable decrease in donor site morbidity [157]. Also, StronBone^®^, a SrO-containing BAG, is available clinically to reduce bone resorption [200]. In general, the use of BAG shows excellent bone repair and reduced donor site morbidity in both long-term and short-term clinical studies [157,200]. Compared to Bioglass^®^ 45S5, FastOs^®^BG resorbs slowly and is more biocompatible and osteoconductive. These properties make the alkali-free BAGs superior alternatives to Bioglass^®^ 45S5 for dental and maxillofacial applications.

Individually customized porous implants of BAG S53P4 and fiber-reinforced composite (FCR) or PMMA (poly(methyl-methacrylate)) as a supporting framework fabricated by additive manufacturing technology have been employed in clinical studies of craniofacial osseous reconstruction with good esthetic and functional outcomes, with follow-up times up to 4 and 5 years [201,202]. This approach did not present any adverse effects or complications and eliminated the donor-site morbidity. BAG-FCR porous cranial implants require intense research as they possess the mechanical strength and biomechanical resemblance to natural bone and are very promising in the future repair of osseous defects [64].

Personalized medicine presents a challenge in terms of adapting the bone substitute to the patient’s specific bone geometry. In contrast to the cranial FCR- or PMMA-BAG implants, scaffolds do not possess mechanical properties of the same degree but are able to deliver drugs or growth hormones for tailored therapeutic purposes [203,204]. A bioactive nanocomposite electroblown scaffold, polycaprolactone, and nanoparticles of BAG, were recently shown to be able to shape and fill defects. Additionally, hDPSCs were successfully added, proliferated, and differentiated into osteogenic cells. Early new bone formation was shown when the scaffold was implanted into alveolar bone defects [205]. Isoniazid and rifampicin against tuberculosis infection were incorporated into 3D-printed scaffolds of chemically modified mesoporous BAG and poly-3-hydroxybutyrate-co-3-hydroxyhexanoate, PHBHHx, for sustained release in bone defects [206,207]. A similar scaffold/template designed to release dimethyloxallyl glycine was investigated using a rat animal model; enhanced angiogenesis and differentiation of bone marrow stem cells were observed [208]. Electrospun and mesoporous BAG scaffolds are the potential treatment approaches for the future, with the possibility of customizing not only the scaffold morphology but also incorporating stem cells, specific drugs, and growth factors for optimizing the individual treatment plans for the patients.

## 6. Conclusions

The chemistry of BAG is mimicking the natural hard tissues composition, and has a bioactive role in the regeneration. BAGs are usually composed of 40–52% SiO_2_, 10–50% CaO, 10–35% Na_2_O, the glass composition may contain 2–8% P_2_O_5_, 0–25% CaF_2_, or 0–10% B_2_O_3_. Na-free BAG prevented the disruption of the glass network and showed equal bioactivity. In addition, various elements such as Si, P, Sr, Cu, F, Ag, Zn, and F are added to enhance the bioactivity and antimicrobial properties. Generally, the BAGs are prepared by either quenching or sol-gel technique. The bioactivity is influenced by the structure and composition of the glass, manufacturing techniques, and the rate of ionic dissolution. The most bioactive glasses have superior surface area with higher dissolution rate and thus faster apatite formation. The FDA has approved Bioglass^®^ 45S5 and S53P4 for clinical applications due to desired antimicrobial properties. There is increasing use of bioactive glasses in various aspects of dentistry including dental restorative materials, toothpaste, mineralizing agents, desensitizing agents, pulp capping, root canal treatment, and air abrasion. Resin composites with BAG and fluoride enhanced dentin remineralization and eliminated enzymatic degradation at the dentin interface. PerioGlas^®^ has been extensively used in periodontal surgical procedures to stimulate bone regeneration, especially in interproximal bone defects due to its hemostatic effect on trabecular bone.
